# Is plasma vitamin C an appropriate biomarker of vitamin C intake? A systematic review and meta-analysis

**DOI:** 10.1186/1475-2891-6-41

**Published:** 2007-11-13

**Authors:** Mahshid Dehghan, Noori Akhtar-Danesh, Catherine R McMillan, Lehana Thabane

**Affiliations:** 1 Population Health Research Institute, School of Medicine, McMaster University, Hamilton, Canada; 2 School of Nursing, McMaster University, Hamilton, Canada; 3 Department of Clinical Epidemiology and Biostatistics, McMaster University, Hamilton, Canada; 4 Division of Plastic Surgery, Sunnybrook Health Sciences Centre, Toronto, Canada; 5 Centre for Evaluation of Medicines, St. Joseph's Healthcare, Hamilton, Canada

## Abstract

**Background:**

As the primary source of dietary vitamin C is fruit and to some extent vegetables, the plasma level of vitamin C has been considered a good surrogate or predictor of vitamin C intake by fruit and vegetable consumption. The purpose of this systematic review was to investigate the relationship between dietary vitamin C intakes measured by different dietary methods and plasma levels of vitamin C.

**Method:**

We searched the literature up to May 2006 through the OVID interface: MEDLINE (from 1960) and EMBASE (from 1988). We also reviewed the reference lists in the articles, reviews, and textbooks retrieved. A total of 26 studies were selected and their results were combined using meta-analytic techniques with random-effect model approach.

**Results:**

The overall result of this study showed a positive correlation coefficient between Food Frequency Questionnaire (FFQ) and biomarker (*r *= 0.35 for "both" genders, 0.39 for females, and 0.46 for males). Also the correlation between Dietary Recalls (DR)/diary and biomarker was 0.46 for "both" genders, 0.44 for females, and 0.36 for males. An overall correlation of 0.39 was found when using the weight record method. Adjusting for energy intake improved the observed correlation for FFQ from 0.31 to 0.41. In addition, we compared the correlation for smokers and non-smokers for both genders (FFQ: for non-smoker *r *= 0.45, adjusted for smoking *r *= 0.33).

**Conclusion:**

Our findings show that FFQ and DR/diary have a moderate relationship with plasma vitamin C. The correlation may be affected/influenced by the presence of external factors such as vitamin bioavailability, absorption condition, stress and food processing and storage time, or by error in reporting vitamin C intake.

## Background

In recent years, a great deal of attention has been given to fruit and vegetable consumption and their role in reducing rates of chronic diseases such as cancer, coronary heart disease (CHD), stroke, diabetes, and arthritis [1-3]. It is suggested that the protective effect of fruits and vegetables is partly due to antioxidant nutrients such as vitamin C and carotenoids which inhibit lipid per-oxidation and oxidative cell damage [4]. However, there is still disagreement on the beneficial effects of ascorbic acid. A large epidemiological study from Finland has found an association between vitamin C intake and reduced risk of death from CHD in women only [5]. Pooled results of 9 cohorts showed reduced incidence of major CHD with high supplemented intake of vitamin C (>700 mg vitamin C/day) [6], with a similar result observed from another study from the UK [7]. On the other hand, the Health Professionals Follow-up Study [8] and the Nurses Health Study [9] did not show significant associations between vitamin C intake and risk of CHD. In a meta-analysis Ness et al. (1999) did not find any significant benefit of vitamin C as a preventive factor for CHD [10].

Vitamin C intake is mainly measured by two different methods: by dietary assessment methods including Food Frequency Questionnaires (FFQ), Diet History Questionnaires (DHQ), 24 hr Dietary Recalls (DR) [11-13], Weight Records (WR), or plasma/serum vitamin C. However, some validation studies used the plasma level of vitamin C as surrogate of vitamin C intake [14,15]. FFQ is one of the most commonly used tools in epidemiologic studies to assess long-term nutritional exposure. It is used to determine usual intakes of selected items from all major food groups. The 24 hr DR and WR are based on recent food intake over a defined period of time with a suitable number of DRs or WRs measured throughout a year, these measurements are considered as an estimation of usual intake [16]. Weight records are widely considered to be the gold standard for dietary intake; however, this method is not feasible in large epidemiological studies. FFQ, documents participants' food intake over the previous year which may result in significant memory error, whereas methods such as DR instruct participants to record the previous day's food consumption and therefore is less prone to memory errors.

As fruits, and to some extent vegetables, are the primary source of dietary vitamin C, the plasma level of vitamin C has been considered a good surrogate or predictor of vitamin C intake by fruit and vegetable consumption. Therefore, significant changes of plasma vitamin C are expected by altering fruit and vegetable consumption. In this meta-analysis, we assessed the association between vitamin C intake measured by dietary assessment methods and plasma level of vitamin C in epidemiological studies. The purposes of this meta-analysis were firstly, to investigate the relationship between dietary vitamin C intakes measured by several different dietary methods and plasma level of vitamin C, secondly, to explore whether the correlation between dietary vitamin C intake and plasma vitamin C varies between different dietary assessment methods.

## Methods

This study was conducted and reported according to the guidelines of meta-analysis of observational studies in epidemiology [17]. We searched the literature up to May 2006 through the OVID interface: MEDLINE (from 1960) and EMBASE (from 1988). We also reviewed the reference lists in the articles, reviews and textbooks retrieved. The search words included: food frequency questionnaire, validation, 24 hour dietary recall, food weight recall, food diaries, vitamin C, ascorbic acid, plasma vitamin C and biomarker. Medical subject heading (MESH) equivalents of the terms were also used. Two independent reviewers (MD and CM) ran the electronic searches and entered the data into Reference Manager 10, removing all duplicates.

Reviewers screened all titles and abstracts for studies that might meet the following inclusion/exclusion criteria:

### Inclusion criteria

This meta-analysis includes cross-sectional or validation studies which published in English language journals and reported either Pearson or Spearman correlations between vitamin C intake measured by dietary assessment methods and plasma level of vitamin C. The Pearson correlation coefficient (*r*) is a measure of the strength of linear association between two approximately normally distributed continuous variables, while the Spearman's correlation coefficient (*ρ*) is a rank-based measure of linear association between two continuous variables where at least one of them is not normally distributed [18].

### Exclusion criteria

Studies were excluded if the dietary intake was not measured quantitatively or if the correlation between dietary intake and serum levels was not reported. We also excluded randomized controlled studies, as the dosages of vitamin C intake were far greater than usual dietary intake. From case-control studies, healthy individuals participated in the control group were included and were also excluded. cases were excluded. Studies published only as abstracts were also excluded.

Studies were also excluded if the age of participants was not between 18–65 years or the participants were pregnant or lactating women, were obese or extra-obese, had a history of diabetes or intestinal digestive disorders, had a diagnosed tumor or were taking anticonvulsants, anticoagulants or a broad spectrum of antibiotics, lipid lowering medications or systemic steroids.

### Outcomes

From each study we extracted the correlation between vitamin C intake and level of plasma vitamin C. Hard copies of potential studies were retrieved and the same two independent reviewers met to reach consensus on the studies to be included. When in doubt, the study authors were contacted; if this was not possible, a third reviewer (NAD) was consulted. Methodological quality was assessed by the same two independent reviewers (unmasked to authors, journals or results). In cases of disagreement, a third reviewer was consulted. We scored the studies using the instrument developed by Dennis et al. [19]. A higher score was assigned to a study if a) the FFQ in the study had a higher number of food items, with an emphasis on fruits and vegetables, b) dietary data was collected by interview-administration, c) the method of conversion of foods to nutrients was defined and the type of nutritional software and food composition tables used in a study was stated, d) more than one dietary assessment tool was used in addition to biomarker measurement, e) the inclusion and exclusion criteria plus the number of excluded participants and the reasons for exclusion were clearly stated, or f) the laboratory assay used to analyze biomarker levels was appropriate and described (e.g. HPLC or colorimetric).

Our main outcome was the correlation coefficient between biomarker and measured dietary vitamin C by different dietary assessment tools; therefore, we assigned more points for studies that a) reported correlations between dietary methods and biomarker for each gender separately and for both genders, b) reported correlations for supplement users and non-supplement users separately, and c) adjusted correlations for energy. Finally, we scored studies based on whether or not quality control was reported. Assessment of study quality was completed using the trial validity assessment sheet. We assessed agreement between reviewers using Cohen's kappa statistics (κ) for categorical variables as poor if κ ≤ 0.2; fair if 0.21 ≤ κ ≤ 0.4; moderate if 0.41 ≤ κ ≤ 0.6; substantial if 0.61 ≤ κ ≤ 0.8; and good if κ ≤ 0.8 [20]. We also used the intra-class correlation coefficient (ICC) to measure the degree of agreement between reviewers for continuous outcomes [21].

Statistical heterogeneity was tested using a chi-square test [22] at alpha = 0.10 and reported with the *I*^2 ^statistic (in which higher values indicate higher heterogeneity). All meta-analyses were carried out using the DerSimonian and Laird [23] random effects model. Subgroup analyses were carried out for each gender based on type of vitamin C measurements (crude, energy adjusted, and supplements excluded: decided a priori). If vitamin C intake was not reported for males and females separately we used "both" as a category to differentiate them from "male" and "female" categories. The statistical package of STATA SE/8 with the "metan" command was used for meta-analysis [24].

### Meta-analysis procedure

We retrieved all effect sizes in the form of Pearson or Spearman correlation coefficients. Following the recommendation by Hunter and Schmidt [25], we did not transform the correlation coefficient into Fisher's z scores as this transformation produces an upward bias in the mean estimation of the correlation coefficients because of the larger weights given to the larger correlations. On the other hand, this upward bias is usually higher than the negligible downward bias produced by untransformed correlations.

## Results

**Figure 1:**
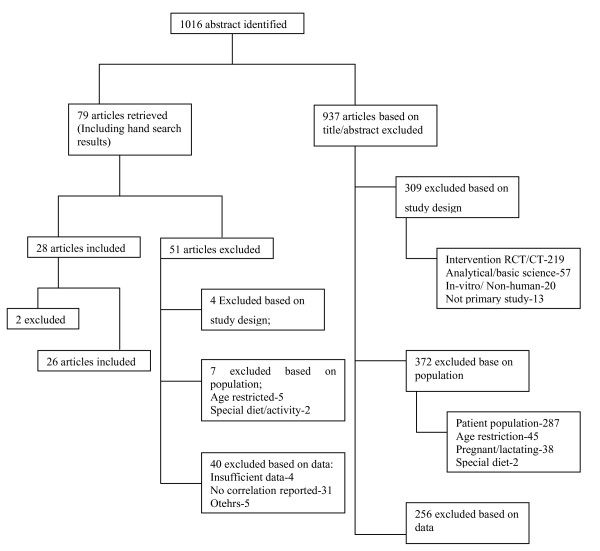
Flowchart for study selection.

The MEDLINE search identified 1016 articles of which 79 articles were potentially relevant. Finally, we chose 26 articles (see Additional file 1) that reported correlation coefficients between plasma levels of vitamin C and dietary intake (see Figure 1). EMBASE provided no more additional studies. One study that reported a negative correlation between dietary intake and plasma level of vitamin C was excluded [26]: a negative relationship between vitamin C intake and plasma vitamin C is biologically implausible. Another study was excluded because of small sample size (n = 5) [27].

### Study characteristics

This analysis included 26 studies with a total of 26631 participants: 18740 participants with DR and diary methods, 6774 with FFQ and 1117 individuals with WR. There were high inter-reviewer agreements between reviewers for inclusion/exclusion criteria and main outcomes: the minimum level of kappa statistics for inclusion/exclusion criteria was 0.86 for FFQ, 0.85 for DR, and 1.0 for WR and the ICC, and 1.0 for main outcome for all methods of FFQ, DR, and WR.

#### Correlation between FFQ and biomarker assessments

**Figure 2:**
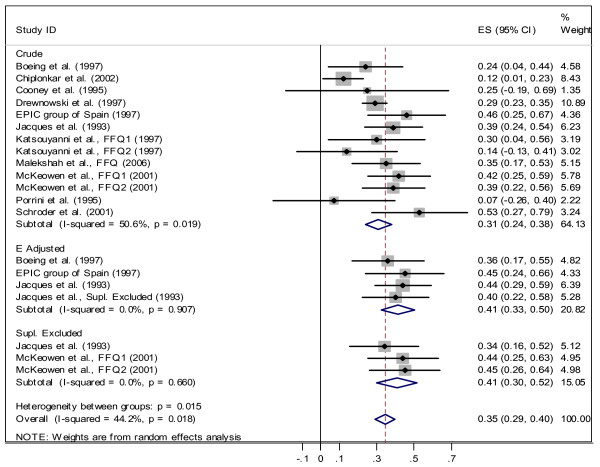
Correlation between dietary vitamin C measured by FFQ and plasma vitamin C for "both" gender.

We found 18 studies with 47 correlation coefficients between FFQ and the biomarker. Some studies reported more than one correlation coefficient for different genders, time-points, or adjusted and unadjusted correlation coefficients based on energy intake, supplement use, and other factors. Twenty correlation coefficients out of these 47 were reported for "both" genders together not for males and females separately. Some studies reported Pearson and some Spearman correlation coefficients. The overall meta-analysis for these 20 correlations resulted in a positive correlation coefficient between FFQ and biomarker (*r *= 0.35; 95%CI: 0.29, 0.40, see Figure 2). This analysis also indicated heterogeneity among correlation coefficients across studies (*χ*^2 ^= 34.02, *df *= 19, *p *= 0.018). The subgroup meta-analysis for "both" genders showed that correlation coefficients for *crude *assessment of vitamin C in FFQs were heterogeneous (*χ*^2 ^= 24.28, *df *= 12, *p *= 0.019); but the correlation coefficients within the two other sub-groups of *energy adjusted *and *supplement excluded *were homogenous (see Figure 2). The higher correlations are seen among studies that reported *energy adjusted *correlations for vitamin C (*r *= 0.41).

**Figure 3:**
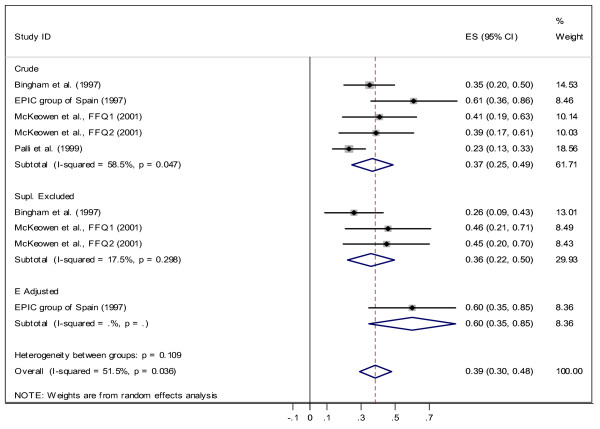
Correlation between dietary vitamin C measured by FFQ and plasma vitamin C for Females.

**Figure 4:**
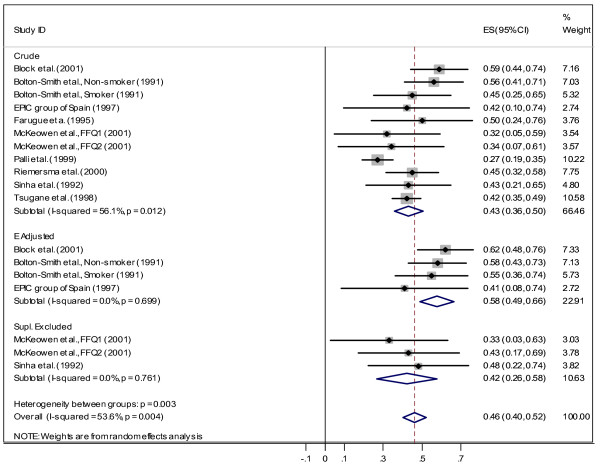
Correlation between dietary vitamin C measured by FFQ and plasma vitamin C for Males.

In the next stage, we carried out the same analysis for each gender separately. The results are presented in Figure 3 and Figure 4. The overall estimated correlation coefficient is 0.46 (CI95%: 0.40, 0.52) for males and 0.39 (CI95%: 0.30, 0.48) for females. The higher correlation coefficients are observed within the *energy-adjusted *group (*r *= 0.60 female, *r *= 0.58 for male). In addition, the correlation coefficients are not homogenous within the *crude *group. For females there is no heterogeneity among subgroups (*χ*^2 ^= 4.43, *df *= 2, *p *= 0.109), but subgroups are heterogeneous for males (*χ*^2 ^= 11.90, *df *= 2, *p *= 0.003). We also evaluated the effect of smoking by comparing correlations between smokers and non-smokers. For non-smokers, the correlation coefficient was 0.39 (CI 95%: 0.26, 0.53) and studies which adjusted for smoking found *r *= 0.33 (CI 95%: 0.23, 0.42). We have found only one study [28] that reported a correlation (*r *= 0.45) for smokers.

Some studies reported correlation coefficient after excluding supplement users from their analyses. For this group we found *r *= 0.41 (CI 95%: 0.3, 0.52) for both genders, *r *= 0.36 (CI 95%: 0.2, 0.50) for female and *r *= 0.42 (CI 95%: 0.26, 0.58) for male.

#### Correlation between dietary recall, weight record and biomarker assessments

**Figure 5:**
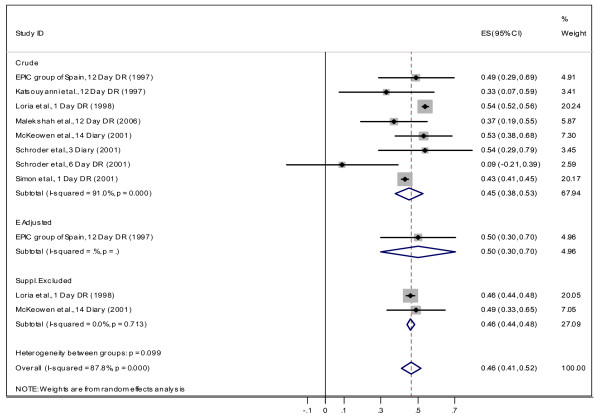
Correlation between dietary vitamin C measured by DR and plasma vitamin C for "both" gender.

**Figure 6:**
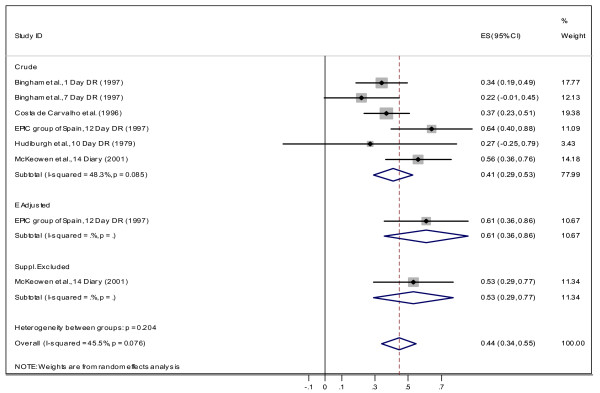
Correlation between dietary vitamin C measured by DR and plasma vitamin C for Females.

**Figure 7:**
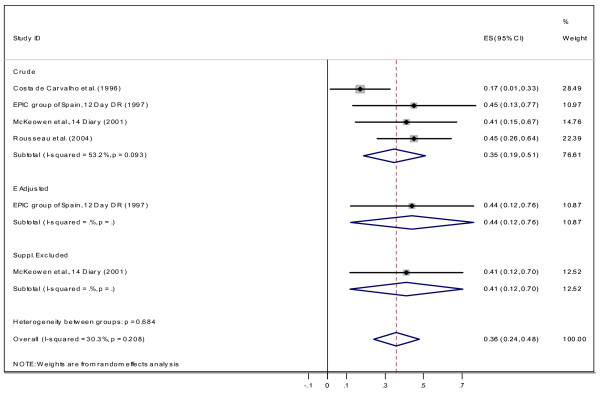
Correlation between dietary vitamin C measured by DR and plasma vitamin C for Males.

We found 10 studies with 25 correlation coefficients, which measured vitamin C using dietary methods such as 24 hr DR or diet diary and biomarker. Again, some studies reported more than one correlation coefficient. Eleven correlations out of 25 were reported for "both" genders together. A meta-analysis on these studies indicates a positive correlation between DR or diet diary and biomarker with *r *= 0.46 (95%CI: 0.41, 0.52, see Figure 5). This analysis indicates homogeneity among groups (*χ*^2 ^= 4.63, *df *= 2, *p *= 0.099) and that correlations within the *crude *group are not homogeneous (*χ*^2 ^= 77.38, *df *= 7, *p *< 0.001). There were only two correlation coefficients in the *supplement-excluded *group. Next, we performed the same meta-analysis for males and females, separately. There was no heterogeneity observed among subgroups (see Figure 6 and Figure 7). The assumption of homogeneity within subgroups was not assessable because there was only one correlation coefficient within *energy adjusted *and *supplement excluded *groups. The estimated correlation coefficient was 0.44 (95% CI: 0.34, 0.55) for females and 0.36 (95% CI: 0.24, 0.48) for males. In our search we found only two studies which reported the correlation of dietary intake and plasma vitamin C for smokers (r = 0.60, 95% CI: 0.50, 0.70) and one study reported the correlation when adjusted for smoking (*r *= 0.36, 95% CI: 0.17, 0.55).

**Figure 8:**
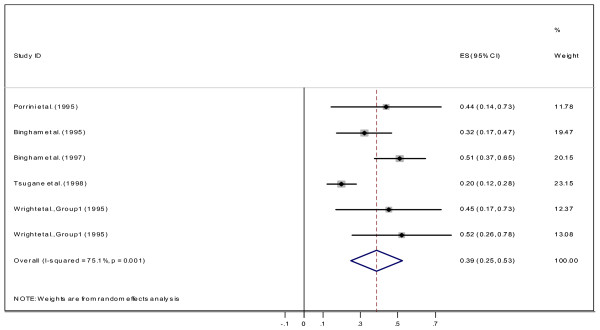
Correlation dietary vitamin C measured by between WR and plasma vitamin C.

Finally, four studies reported a correlation between biomarker and WR method; the overall estimate of the correlation was 0. 39 (CI95%: 0.25, 0.53; Figure 8). No subgroup analysis was carried out because of scarcity of correlation coefficients in this category.

## Discussion

The main goal of the present systematic review and meta-analysis was to investigate the strength of the correlation between plasma level of vitamin C as a biomarker and dietary vitamin C intake measured by methods commonly used for dietary assessment in epidemiological studies. The overall result of this study showed a positive correlation coefficient between FFQ and biomarker (with correlation of 0.35 for "both" genders, 0.39 for females, and 0.46 for males) and between DR/diary and biomarker (with correlation of 0.46 for "both", 0.44 for females, and 0.36 for males). Therefore, for the "both" gender, plasma vitamin C can explain only 12% (*r*^2 ^≅ 0.12) of the variation of vitamin C intake measured by FFQ and 21% (*r*^2 ^= 0.21) measured by DR/diary methods. When comparing correlation coefficients between plasma level and intake (measured by FFQ and DR/diary), we observed a stronger correlation between plasma level and intake measured by DR/diary in women, while correlation between plasma level and intake measured by FFQ was stronger among men (DR: 0.44 for women and 0.36 for men; FFQ: 0.39 for women and 0.46 for men). Adjusting for energy intake also improved the observed correlation for FFQ: crude *r *= 0.31 changed to 0.41. The same effect was also observed for males (crude *r *= 0.43 changed to 0.58). Comparison between vitamin C intake measured by FFQ and plasma level of vitamin C among smoker and non-smoker showed higher correlation for non-smokers (*r *= 0.45) which indicates that even among non-smokers about 20% of variation can be explained by plasma vitamin C. Similar results observed for supplement excluded group (for "both"*r *= 0.41, 17%, for female *r *= 0.36 and for male *r *= 0.42).

The literature indicates that the correlation between vitamin C intake and plasma vitamin C improves after adjusting for certain variables and it becomes evident that the diet-plasma relationship may be influenced by the presence of various confounding factors, such as body size, smoking, the use of supplements, bioavailability, multiple sources of nutrients, food processing techniques, and disease status. These factors may inhibit or enhance absorption and affect nutrient circulating concentration [29]. Participants for the present meta-analysis were <65 years old, although it has been suggested that repletion and depletion of vitamin C is not age dependent [30].

It has been suggested that for saturation of body pool (neutrophils, leukocytes and other tissues) consumption of 100 mg/day of ascorbic acid is adequate [31] and the slope of relation between intake and blood quickly plateau for more intake. As can be seen from Table 1, measured vitamin C intakes by FFQ or other dietary methods are not substantially different from this recommendation (varied from 37 to 327 mg/d). Therefore, the plasma level of participants was not at plateau level.

**Table 1:** BMI (or weight), smoking status, and vitamin C intake of participants involved in studies included in the meta-analysis

			**Measured Vitamin C intake (mg/d)**	
**Study**	**BMI/Weight (kg)**	**Smoking Status**	**DR/WR/FR**	**FFQ**

**1. Bingham **et al., 1995 [12]	NR	NR	87 (56)	122 (53)
Taken from 1994				
**2. Block **et al., 2001 [33]	Wt = 82.6 (11.0)	non-smoker	NR	NR
**3. Boeing **et al., 1997 [34]	NR	adjusted for smoking	NR	NR
**4. Bolton-Smith **et al., 1991 [28]		non-smoker		
both gender	25.8 (3.2)			61.1 (24.8)
**5. Chiplonkar **et al., 2002 [35]		NR	NR	NR
Men	BMI = 20.8 (3.3)			
Women	BMI = 20.8 (3.9)			
**6. Costa de Carvalho **et al., 1996 [36]		NR		
Men	BMI = 24.8 (3.5)		88 (79)	
Women	BMI = 22.4 (3.4)		83 (77)	
**7. Cooney **et al., 1995 [37]	NR	Non-smoker	NR	NR
**8. Drewnowski **et al., 1997 [15]		NR	NR	NR
Men	BMI = 24.4			
Women	BMI = 22.6			
**9. EPIC group of Spain**, 1997 [38]				DH
Men	NR	NR	100.9 (50.1)	116.4 (86)
Women			110.8 (57)	121.9 (72.3)
**10. Faruque **et al., 1995 [39]				
both gender	20.0 (0.4)	Non-smoker	83.4 (7.5)	------
**11. Hudiburgh **et al., 1979 [40]				
Women	NR	NR	140 (148)	------
**12. Jacques **et al., 1993 [41]				
Men	NR	NR	------	256 (154)
Women				221 (154)
**13. Katsouyanni **et al., 1997 [42]				
Men		non-smoker	170.3 (59.1)	304.8 (145.5)
Women			145.7 (44.7)	327.5 (170.1)
**14. Lori **et al., 1998 [43]				
Men			73.1	
Women			75.2	
**15. Malekshah **et al., 2006 [44]				
Men	24.0 (3.9)	NR	45 (21)	89 (54)
Women	26.5 (5.8)		37 (24)	69 (42)
**16. McKeowen **et al., 2001 [45]				
Men	27 (3)	NR	77 (34)	111 (41)
Women	25 (4)		95 (48)	132 (65)
**17. Palli **et al., 1999 [46]	*	adjusted for smoking		
Men			------	92.7 (2.0)
Women				95.4 (2.2)
**18. Porrini **et al., 1995 [47]	21.1 (2.7)	Non-smoker		
both gender			86.4 (39.7)	108.7 (49.2)
**19. Riemersma **et al., 2000 [48]	26.1 (0.3)	adjusted for smoking	------	54.8 (1.8)
**20. Rousseau **et al. 2004 [49]	2.4 (2.5)	Non-smoker	------	
**21. Schroder **et al., 2001 [50]				
both gender	22.4 (2.9)	8% smoker	105 (100)	267 (182)
**22. Simon **et al., 2001 [2]	25.5–26.6	Adjusted	------	
**23. Sinha **et al., 1992 [51]	Wt = 80.9 (1.34) kg	Non-smoker	------	155 (11.0)
**24. Tusgane **et al., 1998 [52]		Adjusted for smoking		55.7
**25. Wright **et al., 1995 [53]	Normal population	Smoker and non-smoker		
Men			66 (5)	
Women			72 (5)	

*317 subject < 23.4, 322 subjects 23.4–26.3, 306 subjects > 26.3

**NR **not reported **NA **not available **DH **dietary history

We removed the effect of sex by conducting separate analyses for males and females. Although we were not able to adjust for fat-free mass (FFM), participants included in these studies had BMI of 20 to 27 and were not obese or underweight. Also, because we conducted a separate analysis for each gender the effect of body size was mostly taken into account. As Blanchard [30] showed, a significant portion of gender difference on vitamin C depletion and repletion is related to FFM effects. Furthermore, we compared the method of analysis among all studies (see Additional file 2), almost all studies followed the same procedure for blood storage and analysis.

For both genders even after energy-adjustment only 36% of variation was explained by plasma vitamin C. An observed moderate correlation may be the consequence of uncontrollable confounding factors or due to inaccurate dietary intake measurement. Also, because of the burden of the data collection process on respondents, accurate measuring of dietary intake may be difficult. In addition, the plasma biomarker measures the amount of vitamin C in foods after storage, preparation, digestion and absorption which may also depend on various homeostatic and metabolic mechanisms.

The observed moderate correlation between the biomarker and intake measured by FFQ may support the suggestion that FFQs are more reliable for ranking the dietary intake of subjects as opposed to determining intake as a primary outcome [32]. While a stronger correlation exists between biomarker and DR, it may be suggested that several days of DR could measure long-term vitamin C intake more accurately [16].

The main limitation of this meta-analysis is that it is based on observational studies; therefore, the confounding factors that might affect the correlation between plasma vitamin C intake and dietary intake could not be controlled.

## Conclusion

Evidently, our findings imply that FFQ and DR/diary and plasma vitamin C have a moderate relationship and therefore, may not measure the same thing. This analysis explains some reasons for the disagreement between intake and plasma level of vitamin C in observational studies. Dietary assessment methods measure dietary intake while plasma vitamin C is a measure of the level of plasma vitamin C circulating in the blood. The relation between intake and plasma level of vitamin C would probably be less complicated if we could assess vitamin C intake more accurately. Although plasma vitamin C level is probably a fair reflection of vitamin C status, many factors, which are mentioned previously, influence this relation.

## List of abbreviations

FFQ Food Frequency Questionnaire

DR Dietary Recalls

MESH Medical subject heading

FFM fat-free mass

## Competing interests

The author(s) declare that they have no competing interests.

## Authors' contributions

MD participated in design of study, ran the electronic searches, reviewed all abstracts and articles, coordinated and drafted the manuscript, revised and approved final manuscript.

NAD participated in design of study, performed statistical analysis, drafted the statistical part of manuscript, took part as the third reviewer, reviewed and approved final manuscript.

CRM ran the electronic searches, reviewed all abstracts and articles, helped to draft the manuscripts, reviewed and approved final manuscript.

LT participated in design of study, helped to draft the manuscripts, reviewed and approved final manuscript.

All authors read and approved the final manuscript.

## Supplementary Material

Additional file 1 Description of studies included in meta-analysis. Studies that reported the correlation coefficient between dietary intake of vitamin C measured by dietary assessment tools and plasma level of vitamin C. Click here for file

Additional file 2 Summary of blood collection and analytical methods. Description of blood collection and analytical method for studies that reported the correlation coefficient between dietary intake of vitamin C measured by dietary assessment tools and plasma level of vitamin C. Click here for file
