# Supplementary material for: Is plasma vitamin C an appropriate biomarker of vitamin C intake? A systematic review and meta-analysis
Source: Nutr J. 2007 Nov 13;6:41. doi: 10.1186/1475-2891-6-41 (PMC2200644; doi:10.1186/1475-2891-6-41)
Supplement: Additional file 1 — Description of studies included in meta-analysis. Studies that reported the correlation coefficient between dietary intake of vitamin C measured by dietary assessment tools and plasma level of vitamin C. [file 1475-2891-6-41-S1.pdf]

Additional file 1 - Description of studies included in meta-analysis

Study	Setting	Sample size & characteristic	Study length /mo.	Diet success rate	Blood / Success rate	No. of FFQ food items	Dietary tool
1. Bingham et al., 1995 [12]	General Practitioners, Cambridge, UK	160 women, aged 50-65 y	12	100%	100%	___	16d WR
2. Bingham et al., 1997 [13]	Cambridge UK	127 women, aged 50-65 y	12	79% for FFQ, 91% for DR, 46% for WR	≅80%	116	FFQ, WR, DR, Diary
3. Block et al., 2001 [33]	Participants of CLUE II study, USA	116 men, aged 35-72 y	NR	NR	NR	60	FFQ
4. Boeing et al., 1997 [34]	EPIC, Germany	43 men, 49 women, aged 35-64 y	12	75%	75%	158	FFQ, 12 DR
5. Bolton-Smith et al., 1991 [28]	General Practitioners, Glasgow, UK	79 non-smoker men, aged 46.0 (2.8)	NR	64.5%	64.5%	65	FFQ
6. Chiplonkar et al., 2002 [35]	Healthy individuals, India	214 men, 108 women, aged 20-50	1	64.4%	64.4%	278	FFQ,
7. Costa de Carvalho et al., 1996 [36]	Free living, France	157 male, 180 female, aged 30-62y	1985-1986	35%	100%	___	7 day food record
8. Cooney et al., 1995 [37]	Free living, Hawaii	13 men, 14 women, aged 31-63 y	12	NR	100%	250	DHQ
9. Drewnowski et al., 1997 [15]	ISTNA survey, Paris	361 men, 476 women, aged >18y	6	100%	100%	73	DH
10. EPIC group of Spain, 1997 [38]	EPIC	32 men, 40 women, aged 35-60 y	12	≅ 80%	79%	___	DH, 12 DRs
11. Faruque et al., 1995 [39]	Students of Dhaka University, Bangladesh	44 men, aged 22-28y	NR	100%	100%	___	7 day food frequency
12. Hudiburgh et al., 1979 [40]	College women, USA	15 women, aged 19-37y	≈ 1	100%	100%	___	7 day food record

Additional file 1 (continued)

13. Jacques et al., 1993 [41]	Cataract case-control, USA	57 men, 82 women, 40-83y	NR	100%	100%	116	FFQ
14. Katsouyanni et al., 1997 [42]	EPIC, Greek component, primary school teachers	42 men, 38 women, aged 25-67y	12	82%	55%	190	FFQ, 12 DRs
15. Lori et al., 1998 [43]	NHANES II, USA	8456 men and women, aged 30-74y	NA	91%	91%	NR	DR
16. Malekshah et al., 2006 [44]	Esophageal cancer cohort, Northern Iran	51 men, 80 women aged 35-65y	12	92%	92%	150	FFQ, 12 DRs
17. McKeowen et al., 2001 [45]	EPIC, Norfolk UK cohort	48 men, 70 women aged 45-74y	9	75%	69%	130	FFQ, 7 day diary, DR
18. Porrini et al., 1995 [46]	Students and technicians, Italy	38 men, women, mean age 27.1 (6.3)	8 days	86%	72%	93	FFQ, 7 day WR
19. Palli et al., 1999 [47]	Healthy control of a case-control study, Italy	553 men, 392 women, aged 30-75y	1985-1988	100%	79%	181	FFQ
20. Riemersma et al., 2000 [48]	Control group of a case-control study, UK	177 men, mean age 53.4 (0.5)	1992-1994	97%	97%	NR	FFQ
21. Rousseau et al. 2004 [49]	Athletes and sedentary individuals, France	Athletes; 84 Men, mean age 26.8 (6.8)	8 days	88%	100%	___	7 day food record
22. Schroder et al., 2001 [50]	Free living, North-eastern Spain	44 men and women, mean age 30.7 (10.4)	≈2	100%	100%	157	FFQ, 72-hr DR
23. Simon et al., 2001 [2]	NH2MS, USA	8453 men and women age 30-75	1976-1980	NR	NR	___	DR
24. Sinha et al., 1992 [51]	Free living, Maryland USA	68 men, aged 30-59 y	NR	100%	100%	60	FFQ

Additional file 1 (continued)

25. Tusgane et al., 1998 [52]	Stomach cancer cross sectional study, Japan	621 men, aged 40-49 y	1 month	98%	96%	34	FFQ, 21 day WR
26. Wright et al., 1995 [53]	Free living, Norway	41 adult male<br>32 elderly male<br>42 adult female<br>42 adult female<br>Adult age: 20-64y<br>Elderly age: 68-73y	1989 to 1991	100%	100%	___	Weight record

NR not reported NA not available DH dietary history
