# Supplementary material for: Is plasma vitamin C an appropriate biomarker of vitamin C intake? A systematic review and meta-analysis
Source: Nutr J. 2007 Nov 13;6:41. doi: 10.1186/1475-2891-6-41 (PMC2200644; doi:10.1186/1475-2891-6-41)
Supplement: Additional file 2 — Summary of blood collection and analytical methods. Description of blood collection and analytical method for studies that reported the correlation coefficient between dietary intake of vitamin C measured by dietary assessment tools and plasma level of vitamin C. [file 1475-2891-6-41-S2.pdf]

Additional file 2 - Summary of blood collection and analytical methods

**Study**	**Fasting blood**	**Blood storage condition and stabilizer**	**Analytical method used**	**Vitamin C concentration** **(**µmol/l)
1. Bingham et al., 1995 [12]	Yes	Within 3-4 hrs. returned to lab in insulated picnic box. Stabilizer; Meta-phosphoric acid. Stored at -40° C.	Liquid chromatography with electrochemical detection	Lower quintile 69.0 (5.0) Middle quintile 62.0 (4.0) Topper quintile 61.0 (5.0)
2. Bingham et al., 1997 [13]	Yes	Within 3-4 hrs. returned to lab in insulated picnic box. Stabilizer; Meta-phosphoric acid. Stored at -40° C.	Liquid chromatography with electrochemical detection	Lower quintile 69.0 (5.0) Middle quintile 62.0 (4.0) l Topper quintile 61.0 (5.0)
3. Block et al., 2001 [33]	NR	Within few hr. returned to lab. Stabilizer; Meta-phosphoric acid. Stored at -70° C.	Spectrophotometrically by using 2,4 dinitrophenylhydrazine	56.8 (22.7)
4. Boeing et al., 1997 [34]	NR	Immediately protected from light. Stabilizer; Meta-phosphoric acid. Stored at -80° C.	Measured by Hoffman LaRoche in Basel	First quintile 7.0 (2.3) Third quintile 7.4 (2.1) Fifth quintile 8.1 (1.5)
5. Bolton-Smith et al., 1991 [28]	NR	Immediately analyzed	NR	Non-smoker 37 (33.1) Smoker 18.4 (17.0)
6. Chiplonkar et al., 2002 [35]	Yes	Within 2 hrs. returned to lab in insulated ice bag.	Spectrophotometer	Men 17.0 (6.3) Women 19.9 (6.8)
7. Costa de Carvalho et al., 1996 [36]	Yes	Sample kept on ice and protected from light. Stored at -30° C	Colorimetric dinitrophenylhydrazin method	Men 64.6 (28.9) Women 76.5 (27.8)
8. Cooney et al., 1995 [37]	Yes	Sample kept at 4° C or 30 min. Stored at -70° C. Stabilizer; Meta-phosphoric acid.	Spectrophotometrically with the use of dichlorophenolindophenol	89.6 (43.0)
9. Drewnowski et al., 1997 [15]	Yes	NR	Automated method based on the continuous flow principle	Age group 30-40y: 49(30) 40-50 y: 42(19) 50-65 y: 51(20)

Additional file 2 (Continued)

10. EPIC group of Spain, 1997 [38]	Yes	Kept from light immediately. Stabilizer; Meta-phosphoric acid. Stored at -180° C.	Microfluorimetric method	Men<br>19.1 (7.1)<br>Women<br>28.8 (8.9)
11. Faruque et al., 1995 [39]	NR	Plasma immediately mixed with Thrichloroacetic acid. Stored at -20° C.	Dinitrophenylhydrazin method	Non-smoker<br>30.1 (1.7)<br>Smoker<br>22.1 (1.1)
12. Hudiburgh et al., 1979 [40]	Yes	NR	Zannoni et al (1974). Micro-modification method which relies on the reduction of ferric iron by ascorbic acid	68.0 (14.8)
13. Jacques et al., 1993 [41]	NR	NR	2,4-dinitrophenylhydrazine method	67 (25)
14. Katsouyanni et al., 1997 [42]	Yes	Plasma stored at -70° C.	HPLC	NR
15. Loria et al., 1998 [43]	½ subjects randomly had fating blood 10-16 hrs.	Shipped on dry ice to lab. Stabilizer; Meta-phosphoric acid	2,4-dinitrophenylhydrazine method	Median (min – Max)<br>51.1 (22.7-73.8)
16. Malekshah et al., 2006 [44]	NR	Kept from light in -8 to +1 ° C for max 2hr. Stabilizer; Meta-phosphoric acid. stored in -80° C	Fluorometric assay	First measurement<br>19.21 (7.8)<br>Second measurement<br>25.68(11.03)
17. McKeowen et al., 2001 [45]	Yes	Taken to lab 3-4 hr after blood withdraw, stored in 4-7° C for 1 week until analyzed. stabilizer; Meta-phosphoric acid	Fluorometric assay	Men:<br>58 (17.5)<br>Women:<br>65.2 (20)
18. Porrini et al., 1995 [46]	Yes	EGTA_Glutathione and protected from light	Enzymatic oxidation of L-AA, condensed with *O*-phenylenediamine . Analyzed with HPLC	56.2 (14.8)
19. Palli et al., 1999 [47]	Yes	Kept from light in4 -8 ° C for max 2hr. Stored at -30° C in lab. stabilizer; Meta-phosphoric acid	Fluorometric assay	38.6 (1.1)
20. Riemersma et al., 2000 [48]	Yes	Stored at -40° C	Enzymatic oxidation	NR
21. Rousseau et al. 2004 [49]	Yes	Immediately protected from light. Stored -80° C. Stabilizer; Meta-phosphoric acid	Fluorimetry using an automated method.	Male:<br>59.3 (13.7)<br>Female:<br>65.6 (13.5)
22. Schroder et al., 2001 [50]	Yes	Homocystein/ 10% trichloroacetic acid add to plasama and stored at -80° C.	Liquid chromatography with electrochemical detection	NR

Additional file 2 (Continued)

23. Simon et al., 2001 [2]	Yes	Stored at -70 to -80° C. stabilizer; Meta-phosphoric acid	HPLC technique	>22.7
24. Sinha et al., 1992 [51]	Yes	Kept on ice for 30 min until processing. Stored at -70 to -80° C. stabilizer; Meta-phosphoric acid	2,4-dinitrophenylhydrazine method	58.5 (2.0)
25. Tusgane et al., 1998 [52]	NR	Frozen after 30 min by dry ice. Stored at -70 to -80° C. stabilizer; Meta-phosphoric acid	Fluorimetrically (iodine oxidation and condensation with 1,2-phenylenediamine)	42.9 (15.0)
26. Wright et al., 1995 [53]	Yes	Placed on dry ice until processing. Stored at -40° C. . stabilizer; Meta-phosphoric acid	HPLC technique	49.4 (4.0)
